# Severe mental illness and substance use disorders in prisoners in low-income and middle-income countries: a systematic review and meta-analysis of prevalence studies

**DOI:** 10.1016/S2214-109X(18)30539-4

**Published:** 2019-03-14

**Authors:** Gergő Baranyi, Carolin Scholl, Seena Fazel, Vikram Patel, Stefan Priebe, Adrian P Mundt

**Affiliations:** a Centre for Research on Environment, Society and Health, Department of Geography, School of GeoSciences, The University of Edinburgh, Edinburgh, UK; b School of Informatics, The University of Edinburgh, Edinburgh, UK; c Department of Psychiatry, University of Oxford, Oxford, UK; d Department of Global Health & Social Medicine, Harvard Medical School, Harvard University, Boston, MA, USA; e Unit for Social and Community Psychiatry, WHO Collaborating Centre for Mental Health Services Development, Queen Mary University of London, London, UK; f Medical Faculty, Universidad Diego Portales, Santiago, Chile; g Medical School, Universidad San Sebastián, Puerto Montt, Chile

## Abstract

**Background:**

Although more than two thirds of the world's incarcerated individuals are based in low-income and middle-income countries (LMICs), the burden of psychiatric disorders in this population is not known. This review provides estimates for the prevalence of severe mental illness and substance use disorders in incarcerated individuals in LMICs.

**Methods:**

For this systematic review and meta-analysis, we searched 17 electronic databases to identify prevalence studies of psychiatric disorders in prison populations in LMICs, published between January, 1987, and May, 2018. We included representative studies from general prison samples, providing information about four major psychiatric diagnoses: psychosis, major depression, alcohol use disorders, and drug use disorders. We pooled data from studies using random-effects meta-analyses and assessed the sources of heterogeneity by meta-regression. We extracted general population estimates from the Global Burden of Diseases 2016 database to calculate comparative prevalence ratios. This study is registered with PROSPERO, number CRD42015020905.

**Findings:**

We identified 23 publications reporting prevalence estimates of severe mental illness and substance use disorders for 14 527 prisoners from 13 LMICs. In this population, the estimated pooled 1 year prevalence rates for psychosis were 6·2% (95% CI 4·0–8·6), 16·0% (11·7–20·8) for major depression, 3·8% (1·2–7·6) for alcohol use disorders, and 5·1% (2·9–7·8) for drug use disorders. We noted increased prevalence at prison intake and geographic variations for substance use disorders. For alcohol use disorders, prevalence was higher in the southeast Asian region than in the eastern Mediterranean region; and drug use disorders were more prevalent in the eastern Mediterranean region than in Europe. Prevalence ratios indicated substantially higher rates of severe mental illness and substance use disorders among prisoners than in the general population (the prevalence of non-affective psychosis was on average 16 times higher, major depression and illicit drug use disorder prevalence were both six times higher, and prevalence of alcohol use disorders was double that of the general population).

**Interpretation:**

The prevalence of major psychiatric disorders is high in prisoners in LMIC compared with general populations. As these findings are likely to reflect unmet needs, the development of scalable interventions should be a public health priority in resource-poor settings.

**Funding:**

CONICYT of the Chilean government and the Wellcome Trust.

## Introduction

More than 7 million prisoners are based in low-income and middle-income countries (LMICs), comprising about 70% of the world's total prison population.^1^ Conditions in these facilities are usually characterised by overcrowding, poor nutrition, and sanitation, and limited or complete lack of access to basic health care, which have raised public health and human rights concerns.^2^, ^3^ However, apart from one review in 2012,^4^ which included only a few studies from LMICs, the prevalence of major psychiatric disorders is not reliably known.^4^, ^5^ Over the past 5 years, several high-quality prevalence studies have been published from LMIC settings.^6^, ^7^

Mental health and substance use disorders are common among people involved with the criminal justice system.^4^, ^8^, ^9^ Although prisoners with unmet mental health-care needs are at higher risk of suicide attemps,^10^ mortality,^11^ and recidivism after release,^12^ mental health disorders often remain undiagnosed and untreated in correctional settings.^3^, ^5^ Up to now, most research on mental health problems in prisoners has focused on high-income countries (HICs). Establishing the prevalence rates of severe mental illness and substance use disorders in LMICs will provide a basis for service and policy developments in countries with resource-poor correctional settings.

We aimed to systematically review the literature of severe mental illness (psychotic disorders and major depression) and substance use disorders (alcohol use disorders and illicit drug use disorders) in prison populations in LMICs, to estimate prevalence rates and prevalence ratios, and to examine sources of heterogeneity.

**Research in context**

**Evidence before this study**

Although 70% of incarcerated men and women are residing in low-income and middle-income countries, almost all evidence on mental disorders among prisoners is based on studies from high-income countries, providing implications that are not applicable or generalisable to poorly resourced settings. The prevalence of psychiatric disorders in the penal justice systems of low-income and middle-income countries (LMICs) is likely to differ from high-income countries because of the scarcity of resources, as well as cultural and legal factors.

To fill this knowledge gap, we systematically searched for prison prevalence studies based in LMICs published between January, 1987, and May, 2018, in 17 electronic global databases, including sources of grey literature. Our search terms covered a range of key words and subject headings on mental health, prison conditions, and epidemiological investigations. We included representative studies from general prison samples from LMICs, providing information about four major psychiatric diagnoses: psychosis, major depression, alcohol use disorders, and drug use disorders, published in any language. Our search identified no systematic reviews focusing on the context of LMICs.

**Added value of this study**

We identified 23 studies from 13 countries, most of which had not previously been included in reviews. Our analysis established the pooled 1 year prevalence rates of four major mental illnesses in prisoner populations in LMICs. Furthermore, our findings emphasise that on arrival to prisons in LMICs, mental disorders may be more prevalent than in samples that also represent later stages of imprisonment.

**Implications of all the available evidence**

In LMICs, the prevalence of psychiatric disorders in prison populations is higher than among people living in the community. Rates in prison populations of LMICs might be even higher than in high-income countries. Because correctional facilities often lack basic health care in low-income and middle-income economies, the implementation of cost-effective interventions and scalable treatments for individuals with mental health problems are needed. Since human rights violations, and physical and psychological abuse are more common in resource-poor correctional settings, protecting the rights and health of people with mental illnesses should be a priority for penal justice policies.

## Methods

This systematic review and meta-analysis was conducted in accordance with the Preferred Reporting Items for Systematic Reviews and Meta-Analyses (PRISMA).^13^

### Search strategy and selection criteria

We conducted a multistage search to identify relevant literature on the prevalence of severe mental illness and substance use disorders in prison populations from LMICs published between January, 1987, and May, 2018. The search strategy comprised a search of online databases (ASSIA; CAB Abstracts; CNKI; Criminal Justice Database; Embase; Global Health; IBSS; LILACS; MEDLINE; NCJRS; PAIS Index; PsycINFO; Scopus; Social Services Abstracts) and the grey literature (Google Scholar; Open Grey; ProQuest Dissertations and Theses Global); screening of reference lists of identified papers and relevant reviews; and corresponding with authors to gain additional information or to clarify data. The appendix provides a full list of the search terms used for the online database searches. Articles from all languages were included.

We included studies in which the following criteria were met: data were collected in general prison populations; the sample was representative for the population of the assessed correctional facility; the study was conducted in a LMIC at the time of data collection or maximum 1 year after classification has changed; the prevalence of severe mental illness and substance use disorders were based on clinical examinations or established with validated questionnaires as part of a clinical or research interview; and diagnoses met the criteria of international diagnostic classifications (Diagnostic and Statistical Manual of Mental Disorders [DSM] or International Classification of Diseases [ICD]). Studies were excluded when: prevalence rates were established in selected subgroups of incarcerated individuals (eg, offender type); sampling strategy was convenient;^14^ data originated from a HIC;^15^ prevalence was reported based on measures and tools that used solely self-report, which did not fulfil diagnostic criteria. Finally, conference abstracts and duplicates were excluded. Two researchers (GB and CS) screened abstracts and full-texts and disagreements between the reviewers were resolved by consensus with APM.

### Data analysis

Two reviewers (GB and CS) independently extracted year and country of data collection, sex, age, type of recruitment (from all prisoners or at admission), sampling strategy, non-response rate, time served in prison, interviewer (mental health professional or research assistant), diagnostic classification system (DSM or ICD), diagnostic instrument, and number of incarcerated individuals, for which 1 year prevalence was reported for psychotic illness (ICD-10 codes: F20–F29, F31, F32·3, F33·3) and major depression (F32–33, except F32·3, F33·3). We extracted both 1 year and lifetime prevalence rates of alcohol (F10) and drug use disorders (F11–19, except F17). Male and female samples were considered separately. Studies that did not report separate rates but included less than 10% of the study participants of one sex were included as representative for the other sex; otherwise they were described as mixed samples. When the year of data collection was not reported, we imputed a year based on the average mean difference between the year of publication and data collection derived from the other studies (4 years).^9^ We prespecified categories for sample size (n<500, n≥500) and average time served in prison (time <1 year, time ≥1 year). Countries were categorised into LMIC and HIC based on their per capita Gross National Income, calculated with the World Bank's Atlas method for the year of data collection. To examine geographic variation of prevalence estimates within LMIC, we used WHO regional classification. If schizophrenia-spectrum, bipolar disorder (which can present with acute psychotic states), and psychotic depression were presented separately, we combined them, in order to create one estimate for overall psychotic disorders. By combining abuse and dependence disorders, we produced single rates for alcohol and drug use disorders.

To assess methodological quality, two reviewers (GB and CS) evaluated the internal and external validity of the included samples based on a modified scale of ten questions,^16^ which allowed a critical appraisal of prevalence rates in epidemiological investigations (appendix).

To account for the heterogeneity between studies, we performed random-effects meta-analysis by estimating the pooled mean of the distribution.^17^ For individual samples, we first calculated 95% score confidence intervals (CIs). Variance of the proportions was stabilised with Freeman-Tukey double arcsine transformation and pooled together with the DerSimonian and Laird method.^18^ The inconsistency between samples was quantified with *I*^2^.^19^ As previous prevalence meta-analyses reported high between-sample heterogeneity, we also provided prevalence ranges.^20^ Sensitivity analysis was conducted pooling 6 month estimates of severe mental illness as reported in a review for HIC.^4^ Pooled rates for subgroups were displayed, when at least five samples were present.

We conducted random-effects meta-regressions by assessing pre-specified sample characteristics on the pooled estimate.^17^ Models in the meta-regression were fitted with the restricted maximum likelihood method and corrected with the Hartung-Knapp variance estimator.^21^ To test whether lower quality investigations systematically distort the pooled estimates, we included the quality score of samples as a covariate. Univariate meta-regression analysis was performed when at least ten samples were available,^22^ multivariate by 20 or more samples, retaining only significant variables (p<0·05).

We calculated prevalence ratios (PR) and their 95% CIs to quantify the difference between the prevalence among prisoners (p) in each sample and in the sex-matched general populations (P) of the respective countries based on the following equation^23^:

$$\left(\text{PR}=\frac{\text{P}}{\text{P}};\text{SE}=\sqrt{\frac{1}{\text{p}\times \text{n}}+\frac{1}{\text{P}\times \text{N}}-\frac{1}{\text{n}}-\frac{1}{\text{N}};}95\%\text{CI}=e^{\ln(\text{PR})\pm 1.96\times \text{SE}}\right)$$

We extracted sex-specific and country-specific prevalence rates from the Global Burden of Diseases 2016 database for the year of data collection in the respective prison survey. The matching population size (N) was imputed from the 2017 Revision of World Population Prospects. Because a national reference for psychosis is not available, rates for schizophrenia were extracted and matched with prison study rates for schizophrenia, if available. If not, we used rates of non-affective psychotic illness. Prevalence ratios were pooled with random-effects meta-analysis. Sensitivity analyses were conducted for studies reporting 6 month rates of severe mental illness; and for schizophrenia, without imputed values of psychotic disorders.

Biased prevalence estimates might arise not only from the inclusion of studies with lower methodological quality but also from publication or small study bias.^22^ To assess publication bias, we drew funnel plots presenting prevalence estimates against their SEs and tested the asymmetry of the funnel plots with Egger's test,^24^ when ten or more samples were available.

All analyses were done with STATA (version 13). This study is registered with PROSPERO, number CRD42015020905.

### Role of the funding source

The funder of the study had no role in study design, data collection, data analysis, data interpretation, or writing of the report. The corresponding author had full access to all the data in the study and had final responsibility for the decision to submit for publication.

## Results

**Figure 1:**
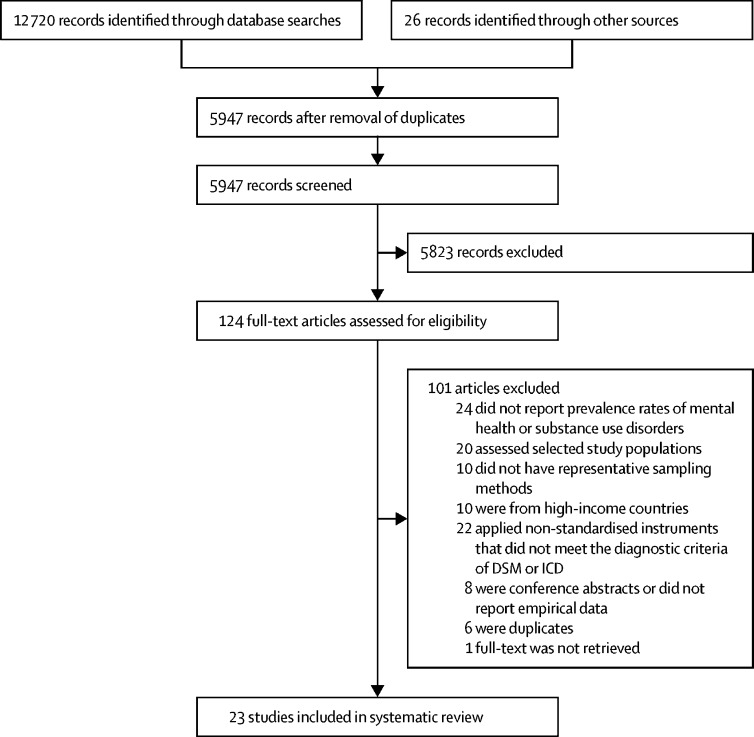
Study identification, screening and eligibility test, following the Preferred Reporting Items of Systematic Reviews (PRISMA) DSM=Diagnostic and Statistical Manual of Mental Disorders. ICD=International Classification of Diseases.

We identified 23 publications with 30 samples published between 1997 and 2018 (figure 1). They provided data from 13 different LMICs: Burkina Faso,^25^ Brazil,^6^, ^26^, ^27^, ^28^ Chile,^7^, ^29^ Egypt,^30^ India,^31^, ^32^, ^33^, ^34^, ^35^ Iran,^36^ Malaysia,^37^ Nigeria,^38^, ^39^ South Africa,^40^ South Sudan,^41^ Sri Lanka,^42^ Togo,^43^ and Turkey.^44^, ^45^ Five studies were written in languages other than English: two in French,^25^, ^43^ two in Portuguese,^27^, ^28^ and one in Turkish.^45^ Of 14 527 imprisoned individuals, 85% were men and the weighted mean age was 31·8 years. Approximately 93% of the participants were prisoners in wards, while 7% at arrival to prison (table 1; appendix).

**Table 1:** Studies reporting prevalence estimates for severe mental disorders or substance use disorders in prison populations of low-income and middle-income countries

	**Country**	**WHO region**	**Sex**	**Sampling**	**Sample size**	**Non-response rate (%)**	**Interviewer**	**Diagnostic instrument**	**Diagnostic criteria**	**Quality appraisal score**
Adesanya et al^38^	Nigeria	Africa	Male	Population	395	4·8	Not stated	Not stated	DSM-III-R	6
Andreoli et al^6^*†	Brazil	Americas	Male	Stratified random	1192	26·8	Trained non-clinician	CIDI	ICD-10	8
Andreoli et al^6^*†	Brazil	Americas	Female	Stratified random	617	10·5	Trained non-clinician	CIDI	ICD-10	9
Assadi et al^36^†	Iran	Eastern Mediterranean	Male	Stratified random	351	12·3	Psychiatrist	SCID-CV	DSM-IV	9
Ayirolimeethal et al^31^‡	India	Southeast Asia	Male	Population	222	3·5	Psychiatrist	MINI-Plus	Not stated	8
Ayirolimeethal et al^31^‡	India	Southeast Asia	Female	Population	33	0·0	Psychiatrist	MINI-Plus	Not stated	7
Boşgelmez et al^44^	Turkey	Europe	Male	Stratified random	30	6·3	Psychiatrist, clinical psychologist	SCID	DSM-IV	7
Boşgelmez et al^44^	Turkey	Europe	Female	Stratified random	30	11·8	Psychiatrist, clinical psychologist	SCID	DSM-IV	7
Canazaro and Argimon^27^	Brazil	Americas	Female	Population	287	22·0	Psychology student, psychologist	SCID-CV	DSM-IV	8
El-Gilany et al^30^†	Egypt	Eastern Mediterranean	Mixed	Stratified random	1350	0·5	Psychiatrist	SCID	DSM-IV	8
Goyal et al^32^	India	Southeast Asia	Male	Random	500	Not stated	Consultant	PSE	ICD-10	7
Joshi et al^35^	India	Southeast Asia	Female	Population	50	Not stated	Psychiatrist	Not stated	DSM-IV TR	6
Kaya et al^45^*‡	Turkey	Europe	Male	Random	305	14·3	Psychiatric assistant, trainee psychiatrist	CIDI	DSM-IV	6
Kumar and Daria^33^	India	Southeast Asia	Male	Random	118	9·2	Psychiatrist	IPIS	ICD-10	7
Majekodunmi et al^39^*‡	Nigeria	Africa	Male	Random	196	1·5	Psychiatrist	SCID	DSM-IV	8
Math et al^34^†	India	Southeast Asia	Male	Population	5024	Not stated	Research assistant	MINI-Plus	Not stated	4
Mundt et al^7^*‡	Chile	Americas	Male	Random	855	1·0	Field worker	CIDI	DSM-IV	9
Mundt et al^7^*‡	Chile	Americas	Female	Random	153	1·0	Field worker	CIDI	DSM-IV	8
Mundt et al^29^	Chile	Americas	Male	Consecutive systematic	229	7·0	Clinical psychologist	MINI	DSM-IV	10
Mundt et al^29^	Chile	Americas	Female	Consecutive	198	7·0	Clinical psychologist	MINI	DSM-IV	9
Naidoo and Mkize^40^	South Africa	Africa	Male	Stratified systematic random	193	22·8	Psychiatrist	MINI	Not stated	7
Nanéma et al^25^‡	Burkina Faso	Africa	Male	Systematic random	419	2·8	Medical student	MINI	ICD-10	6
Ndetei et al^41^†‡	South Sudan	Africa	Mixed	Population	192	53·5	Clinical psychologist	MINI-Plus	ICD-10	5
Niriella et al^42^	Sri Lanka	Southeast Asia	Male	Random	325	0·8	Trained research assistant	Not stated	ICD-10	7
Niriella et al^42^	Sri Lanka	Southeast Asia	Female	Random	68	0·8	Trained research assistant	Not stated	ICD-10	6
Pondé et al^26^	Brazil	Americas	Male	Random; population	497	4·0	Medical student	MINI-Plus	DSM-IV	7
Salifou et al^43^‡	Togo	Africa	Female	Population	61	9·0	Psychiatrist, psychologist	Clinical Interview	DSM-V	7
Silva et al^28^‡	Brazil	Americas	Male	Consecutive	466	3·0	Not stated	MINI-Plus	DSM-IV	7
Silva et al^28^‡	Brazil	Americas	Female	Consecutive	91	3·0	Not stated	MINI-Plus	DSM-IV	6
Zamzam and Hatta^37^†	Malaysia	Western Pacific	Female	Population	80	3·6	Trainee psychiatrist	CIDI	Not stated	7

CIDI=Composite International Diagnostic Interview. DSM=Diagnostic and Statistical Manual of Mental Disorders. ICD=International Classification of Diseases. IPIS=Indian Psychiatric Interview Schedule. MINI=Mini-International Neuropsychiatric Interview. PSE=Present State Examination. SCID=Structured Clinical Interview for DSM Disorders.

* Results are based on 1 year coverage.

† Study reported separate rate for schizophrenia.

‡ Authors provided additional data.

**Figure 2:**
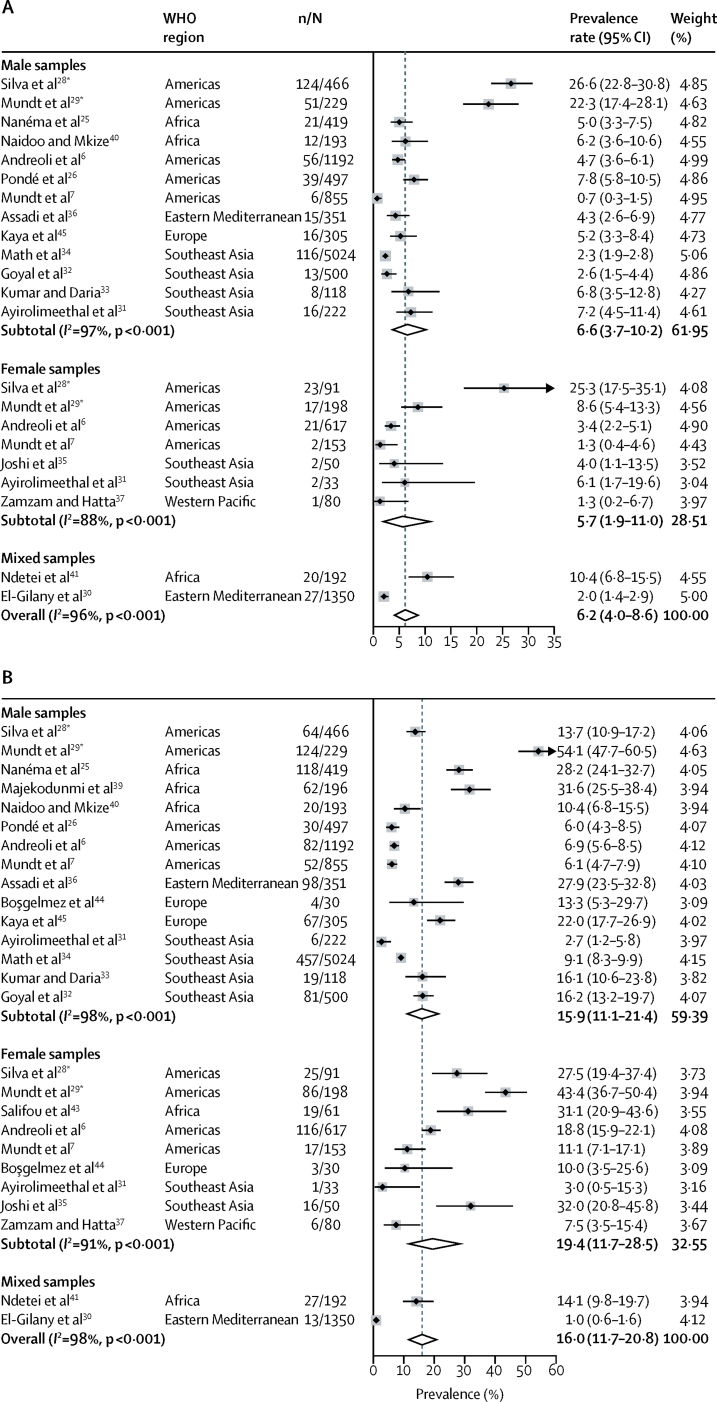
Random-effects meta-analyses of 1-year prevalence studies reporting psychotic disorders (A) and major depression (B) in prison populations in low-income and middle-income countries *Samples were recruited at intake to prison.

1 year prevalence rates of psychotic disorders were reported in 22 samples involving 13 135 individuals.^6^, ^7^, ^25^, ^26^, ^28^, ^29^, ^30^, ^31^, ^32^, ^33^, ^34^, ^35^, ^36^, ^37^, ^40^, ^41^, ^45^ The random-effects pooled prevalence was 6·2% (95% CI 4·0–8·6) with very high between-sample heterogeneity (*I^2^*=96; p<0·001; figure 2). We noted 15·8 times (95% CI 8·7–28·9) higher rates of non-affective psychosis than in the general population (table 2). Meta-regression indicated lower prevalence of psychosis in studies with smaller sample sizes (β^2^=–0·076; p=0·004), decreasing rates with longer time spent in prison (β^2^=–0·146; p<0·001), and higher estimates in samples recruited at prison intake (β^2^=0·186; p<0·001). In the multivariate model, only the elevated prevalence of admission samples remained significant (β^2^=0·138; p=0·026; appendix). The pooled prevalence of psychosis was 3·9% (95% CI 2·8–5·8) in non-admission samples. For this subgroup, prevalence rates ranged from 0·7% to 10·4% with substantial heterogeneity (*I*^2^=87; p<0·001) and were slightly higher in male (4·3%; 95% CI 2·9–6·0) than in female populations (2·5%; 1·5–3·7; data not shown). In the four admission samples,^28^, ^29^ the prevalence varied between 8·6% and 26·6%.

**Table 2:** Prevalence ratios of severe mental illness in prison populations in low-income and middle-income countries

		**Study**	**Sex**	**Psychotic disorders**			**Major depression**		
				Population prevalence	Prevalence ratio		Population prevalence	Prevalence ratio	
					Estimate	95% CI		Estimate	95% CI
Africa									
	Burkina Faso	Nanéma et al^25^	Men	0·12	41·67*	27·48–63·28	1·48	19·05	16·35–22·20
	Nigeria	Majekodunmi et al^39^	Men	..	..	..	1·74	18·16	14·78–22·32
	South Africa	Naidoo and Mkize^40^	Men	0·19	24·74*	13·10–46·70	2·21	4·71	3·11–7·12
	South Sudan	Ndetei et al^41^	Mixed	0·13	32·31	16·44–63·50	1·97	7·16	5·05–10·15
	Togo	Salifou et al^43^	Women	..	..	..	2·45	12·69	8·74–18·44
Americas									
	Brazil	Andreoli et al^6^	Men	0·22	8·64	5·74–12·99	1·95	3·54	2·87–4·36
	Brazil	Pondé et al^26^	Men	0·22	27·27*	19·26–38·63	2·02	2·97	2·10–4·21
	Brazil	Silva et al^28^†	Men	0·22	120·91*	103·98–140·60	1·95	7·03	5·59–8·82
	Brazil	Andreoli et al^6^	Women	0·20	7·50	3·96–14·22	4·26	4·37	3·70–5·15
	Brazil	Silva et al^28^†	Women	0·20	126·50*	88·87–180·07	4·26	6·46	4·62–9·01
	Chile	Mundt et al^7^	Men	0·23	3·04*	1·37–6·76	2·13	2·86	2·20–3·73
	Chile	Mundt et al^29^†	Men	0·23	96·96*	76·10–123·52	2·16	25·05	22·23–28·22
	Chile	Mundt et al^7^	Women	0·21	6·19*	1·56–24·63	3·79	2·93	1·87–4·59
	Chile	Mundt et al^29^†	Women	0·22	39·09*	24·82–61·57	3·61	12·02	10·25–14·10
Eastern Mediterranean									
	Iran	Assadi et al^36^	Men	0·18	11·11	5·34–23·11	3·15	8·86	7·49–10·48
	Egypt	El–Gilany et al^30^	Mixed	0·18	4·44	2·45–8·05	2·28	0·42	0·25–0·72
Europe									
	Turkey	Boşgelmez et al^44^	Men	..	..	..	2·05	6·49	2·60–16·18
	Turkey	Kaya et al^45^	Men	0·19	5·26*	1·72–16·08	2·02	10·88	8·80–13·44
	Turkey	Boşgelmez et al^44^	Women	..	..	..	3·66	2·73	0·93–7·99
Southeast Asia									
	India	Ayirolimeethal et al^31^	Men	0·24	28·33*	17·41–46·11	1·82	1·48	0·67–3·27
	India	Goyal et al^32^	Men	0·23	1·74	0·44–6·94	1·91	8·48	6·95–10·35
	India	Kumar and Daria^33^	Men	0·23	14·78	5·65–38·68	1·90	8·47	5·61–12·79
	India	Math et al^34^	Men	0·24	4·58	3·53–5·96	1·82	5·00	4·58–5·46
	India	Ayirolimeethal et al^31^	Women	0·23	13·04*	1·87–90·78	2·64	1·14	0·16–7·91
	India	Joshi et al^35^	Women	0·23	17·39*	4·47–67·62	2·62	12·21	8·15–18·30
Western Pacific									
	Malaysia	Zamzam and Hatta^37^	Women	0·26	5·00	0·74–33·75	1·57	4·78	2·21–10·31
Pooled prevalence ratio I		..	Total	*I*^2^=97%	15·83	8·68–28·87	*I*^2^=98%	5·95	4·41–8·03
Pooled prevalence ratio II (non–admission samples)		..	Men	*I*^2^=93%	11·10	6·05–20·37	*I*^2^=97%	6·30	4·35–9·13
Pooled prevalence ratio II (non–admission samples)		..	Women	*I*^2^=0%	8·26	5·03–13·58	*I*^2^=89%	5·26	3·10–8·93
Pooled prevalence ratio II (non–admission samples)		..	Total	*I*^2^=90%	10·68	6·68–17·06	*I*^2^=97%	5·31	3·94–7·19

* Admission samples.

† Sample reported non-affective psychotic disorders; otherwise, prevalence of schizophrenia was extracted. Population prevalence refers to the sex-specific, country-specific, and year-specific rates in the general population retrieved from the Global Burden of Disease database 2016.

We identified 26 samples reporting 1 year prevalence of major depression (n=13 452).^6^, ^7^, ^25^, ^26^, ^28^, ^29^, ^30^, ^31^, ^32^, ^33^, ^34^, ^35^, ^36^, ^37^, ^39^, ^40^, ^41^, ^43^, ^44^, ^45^ The pooled 1 year prevalence was 16·0% (95% CI 11·7–20·8) with substantial heterogeneity (*I^2^*=98%; p<0·001; figure 2), indicating 6·0 times (95% CI 4·4–8·0) higher rates than in the general population (table 2). Meta-regression found increased prevalence of major depression at admission (β^2^=0·199; p=0·005) and lower estimates in larger samples (β^2^=–0·116; p=0·039), of which only higher prevalence at prison intake remain significant in the multivariate model (β^2^=0·168; p=0·017; appendix). The pooled estimate of major depression in non-admission samples was 13·2% (95% CI 9·5–17·4). For these individuals, prevalence varied from 1·0% to 32·0%, with very high heterogeneity (*I*^2^=97%; p<0·001), and averaged 13·8% (95% CI 9·7–18·4) in men and 15·2% (9·2–22·4) in women. At prison intake,^28^, ^29^ the estimates ranged between 13·7% and 54·1%.

Findings of our sensitivity analysis on non-admission samples showed no significant variation in prevalence rates or prevalence ratios for severe mental illness in samples reporting only 6 month estimates. The prevalence ratio for samples reporting solely schizophrenia was 7·9 (95% CI 4·9–12·7) compared with the general population (appendix).

For substance use disorders, we considered admission and non-admission samples separately because the former were likely to be higher and more comparable to the literature coming from HIC.^8^ At prison intake,^28^, ^29^ the 1 year prevalence of alcohol use disorders ranged from 13·6% to 42·3%, and for drug use disorders estimates were between 27·3% and 68·1%.

**Figure 3:**
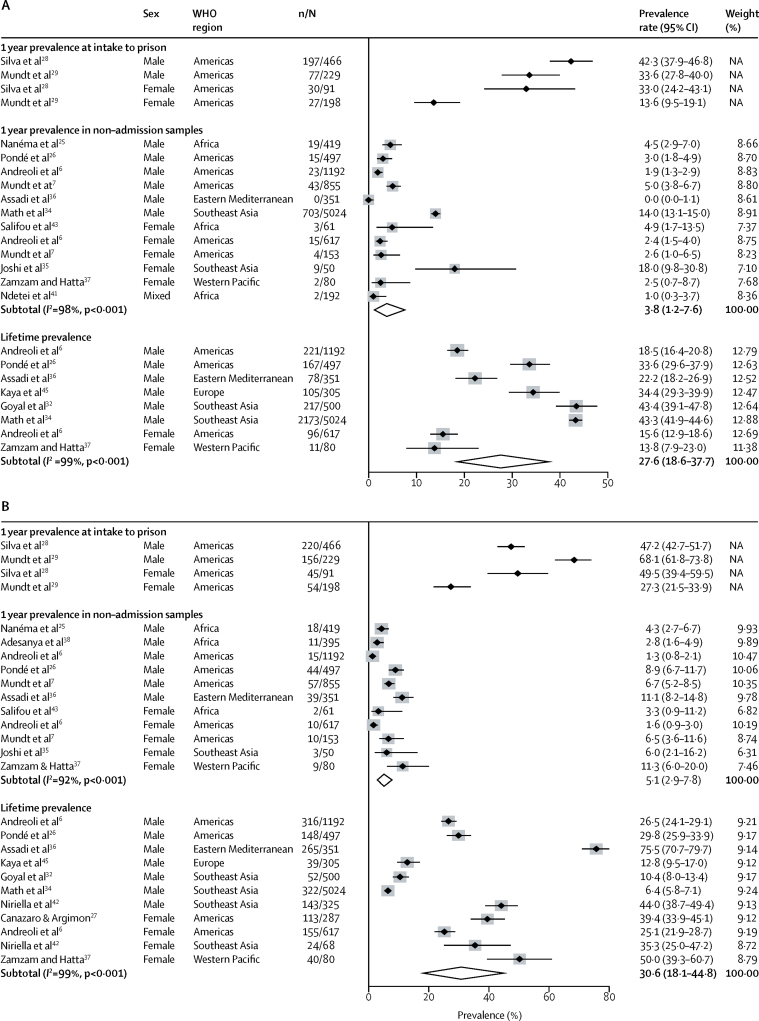
Random-effects meta-analysis of prevalence studies reporting alcohol use disorders (A) and drug use disorders (B) in prison populations in low-income and middle-income countries NA=not applicable.

We identified 12 non-admission samples reporting 1 year prevalence of alcohol use disorders (n=9491).^6^, ^7^, ^25^, ^26^, ^34^, ^35^, ^36^, ^37^, ^41^, ^43^ The pooled prevalence was 3·8% (95% CI 1·2–7·6; figure 3), 2·4 times higher than (1·1–5·2) in the general population (table 3). The estimates ranged from 0·0% to 18·0% (*I*^2^=98%, p<0·001), and were similar for men (3·7%, 95% CI 0·5–9·4) and women (4·4%, 1·5–8·4; figure 3). Meta-regression indicated geographical variation, with elevated prevalence in the southeast Asian region in comparison to the eastern Mediterranean region (β^2^=0·140; p=0·038; appendix). We recorded higher estimates in lower quality studies (β^2^=–0·024; p=0·001), which could be attributed to two lower quality studies with high prevalence estimates from the southeast Asian region.^34^, ^35^ The lifetime prevalence rate of alcohol use disorders (eight samples; n=8566)^6^, ^26^, ^32^, ^34^, ^36^, ^37^ was 27·6% (95% CI 18·6–37·7; men: 32·2%, 22·3–43·0, and women: 15·2%, 12·6–18·0) and varied between 13·8% and 43·4% (*I*^2^=99%, p<0·001; figure 3). The small number of samples precluded further analyses.

**Table 3:** Prevalence ratios of substance use disorders in prison populations in low-income and middle-income countries

		**Study**	**Sex**	**Alcohol use disorders**			**Drug use disorders**		
				Population prevalence	Prevalence ratio		Population prevalence	Prevalence ratio	
					Estimate	95% CI		Estimate	95% Cl
Africa									
	Burkina Faso	Nanéma et al^25^	Men	1·00	4·50	2·90–7·00	0·39	11·03	7·02–17·32
	Nigeria	Adesanya et al^38^	Men	..	..	..	0·37	7·57	4·23–13·53
	South Sudan	Ndetei et al^40^	Mixed	1·11	0·90	0·22–3·68	..	..	..
	Togo	Salifou et al^43^	Women	0·96	5·10	1·69–15·42	0·30	11·00	2·83–42·80
Americas									
	Brazil	Andreoli et al^6^	Men	4·28	0·44	0·30–0·67	1·30	1·00	0·61–1·64
	Brazil	Pondé et al^26^	Men	4·29	0·70	0·42–1·15	1·27	7·01	5·29–9·28
	Brazil	Silva et al^28^*	Men	4·28	9·88	8·89–10·99	1·30	36·31	32·98–39·97
	Brazil	Andreoli et al^6^	Women	1·38	1·74	1·05–2·88	0·72	2·22	1·20–4·13
	Brazil	Silva et al^28^*	Women	1·38	23·91	17·84–32·05	0·72	68·75	55·87–84·61
	Chile	Mundt et al^7^	Men	3·78	1·32	0·99–1·77	1·38	4·86	3·78–6·24
	Chile	Mundt et al^29^*	Men	3·60	9·33	7·78–11·20	1·44	47·29	43·27–51·68
	Chile	Mundt et al^7^	Women	1·46	1·78	0·68–4·70	0·78	8·33	4·57–15·20
	Chile	Mundt et al^29^*	Women	1·40	9·71	6·84–13·80	0·80	34·13	27·18–42·84
Eastern Mediterranean									
	Iran	Assadi et al^36^	Men	0·64	0·22	0·01–3·58	2·50	4·44	3·30–5·97
Southeast Asia									
	India	Math et al^34^	Men	2·03	6·90	6·44–7·39	..	..	..
	India	Joshi et al^35^	Women	0·43	41·86	23·17–75·64	0·37	16·22	5·41–48·58
Western Pacific									
	Malaysia	Zamzam and Hatta^37^	Women	0·32	7·81	1·99–30·70	0·54	20·83	11·26–38·56
Pooled prevalence ratio (non–admission samples)		..	Men	*I*^2^=99%	1·40	0·45–4·36	*I*^2^=92%	4·85	2·93–8·04
Pooled prevalence ratio (non–admission samples)		..	Women	*I*^2^=94%	5·54	1·23–24·92	*I*^2^=86%	8·98	3·62–22·27
Pooled prevalence ratio (non–admission samples)		..	Total	*I*^2^=97%	2·43	1·12–5·24	*I*^2^=89%	6·11	3·98–9·39

* Admission samples. Population prevalence refers to the sex-specific, country-specific, and year-specific rates in the general population retrieved from the Global Burden of Disease database 2016.

For the 11 samples reporting 1 year prevalence rates of drug use disorders (n=4670),^6^, ^7^, ^25^, ^26^, ^35^, ^36^, ^37^, ^38^, ^43^ the pooled estimate was 5·1% (95% CI 2·9–7·8), 5·3% (2·5–9·0) in male and 5·0% (1·6–9·8) in female samples—ie, 6·1 times (95% CI 4·0–9·4) higher than in the general population (table 3). The 1 year prevalence of drug use disorders ranged from 1·3% to 11·3% (*I*^2^=92%; p<0·001; figure 3). Findings of meta-regression did not show any significant explanation for heterogeneity (appendix). Studies on lifetime prevalence of drug use disorders (11 samples; n=9246)^6^, ^26^, ^27^, ^32^, ^34^, ^36^, ^37^, ^42^ indicated a pooled estimate of 30·6% (95% CI 18·1–44·8; men: 27·2%, 95% CI 12·1–45·7, and women: 36·7%, 95% CI 25·9–48·2), ranging between 6·4% and 75·5% (*I*^2^=99%; p<0·001; figure 3). Meta-regression results showed geographical variation between samples with elevated prevalence in the eastern Mediterranean in comparison to the European region (β^2^=0·627; p=0·019; appendix).

Egger's test of asymmetric funnel plot indicated small sample bias for psychotic illnesses (p=0·027), current alcohol use disorders (p=0·025) and for lifetime drug use disorders (p=0·013) in non-admission studies. After excluding the study with the lowest quality score, which also had the largest sample size,^34^ evidence for publication bias did not remain significant (appendix).

## Discussion

Our findings suggest that incarcerated individuals in LMICs have a higher prevalence of psychiatric disorders than the general population and that rates at arrival to prison are elevated. Furthermore, our results show that there is geographical variation in the prevalence of substance use disorders.

The study had several limitations. Our findings are based on only 13 of more than 100 LMICs, and we could not identify any studies meeting our criteria from China, which has the largest prison population among LMICs. Additionally, there was high heterogeneity between studies. This was not unexpected as the included countries are substantially different in terms of their criminal and health-care systems.

Consistent with systematic reviews from prisoners in HICs,^4^, ^8^ our findings provide evidence for higher prevalence of psychiatric disorders in incarcerated people than in the general population.^46^, ^47^ Imprisoned individuals often have a low socioeconomic background, belong to minority groups, and have histories of childhood victimisation and substance abuse, which make them vulnerable to psychiatric disorders.^9^, ^48^ While in prison, poor living conditions,^3^ physical assault^20^ and psychological abuse^5^ can further contribute to mental health disorders.

Although general population reviews indicate a lower prevalence of schizophrenia^47^ and major depression^46^ in LMICs than in HICs, we did not find this among prisoners.^4^ A high prevalence of severe mental illness in prisoners in LMICs could relate to poorly developed community mental health-care systems that do not yet reach socially deprived and marginalised populations in these countries. Human rights violations among individuals with mental health problems during imprisonment, especially for those with psychotic conditions, have been reported to be more common in poorly resourced settings.^5^

Upon arrival to prison, we found similar 1 year prevalence estimates of alcohol and drug use disorders as those reported for individuals in HICs.^8^ These are comparable to lifetime rates and provide information about the substance use problems before imprisonment. However, the estimates on current prevalence among non-intake samples represent the average disease burden during imprisonment, which might be relevant for service planning. Even though addictive substances are available in most prisons in LMICs,^48^ the prevalence of substance use disorders for this population is substantially lower during imprisonment than for the same population while outside of prison. We found regional variation in the prevalence of substance use disorders, possibly linked to regional differences of the substances used.^48^ The highest rates of alcohol use disorders were found in studies from India,^34^, ^35^ while the highest rate for drug use disorders was reported in a study from Iran.^36^ While lower rates of substance use disorders in women are found in the general population,^46^ this is typically not the case for prison populations. The rates of substance use disorders among prisoners start considerably higher than population comparisons independent of sex, likely due to substance use being a major driver of criminality.^49^ In HICs, incarcerated women have similar rates of alcohol use disorders as incarcerated men and a higher prevalence of illicit drug use disorders than men.^8^ This difference can be explained by lower rates of female incarceration and hence women in prison being a more selected group of high-risk individuals with elevated rates of substance use problems.

Admission studies indicated higher rates of psychosis and major depression at arrival to prison compared with investigations that included prisoners at later stages of imprisonment, which is consistent with longitudinal studies from HICs reporting high rates of psychiatric disorders at intake to prison.^50^, ^51^ However, this finding was based on only two intake studies conducted in Latin American countries. The very high prevalence of severe mental illness at intake to prison in those countries could be linked to the use of cocaine products before imprisonment.^29^, ^48^, ^51^ There are several possible explanations for lower rates of mental health symptoms at later stages of imprisonment in spite of the harsh conditions of LMICs prisons including: reduced access to substances during imprisonment, protection or removal from adverse social environments outside of prisons, development of coping mechanisms,^50^ some availability of treatment services, and diversion of mentally ill prisoners.^3^ However, the literature points to substantial unmet health-care needs.^3^

Our findings have several implications. First, the low number of included samples emphasises the paucity of epidemiological investigations in LMICs. Although more than 100 high quality samples provide reliable evidence of psychiatric disorders in prisons in HICs,^20^ we found only 30 samples from a much more diverse group of countries. Further evidence is needed to adequately plan interventions for prisoners with mental disorders in LMICs, especially from regions underrepresented in research such as central and east Asia, and Central America. Second, cost-effective interventions and scalable treatments should be prioritised, either by adapting existing programmes from HICs to local conditions or by developing new programmes on a large scale (eg, interventions at the transition from prison to the community for individuals with mental illness).^52^, ^53^ Effective psychological treatments in prison settings have been reported for HICs^52^ and some might be transferable to resource-poor settings. Furthermore, community interventions in LMICs, such as enhancing health literacy,^54^ using digital technologies in prevention,^55^ as well as treatments of severe mental disorders,^56^ have shown promising ways of addressing the mental health treatment gap. Some of these interventions could also be used to prevent and treat psychiatric disorders in prison populations.

Finally, imprisonment could present an opportunity to treat people with mental health and substance use problems who otherwise would be difficult to reach for health services;^4^ however, neither the funding nor qualified staff for such treatments are usually available in prisons. National governments in LMICs should move the responsibility for prison health care from prison administrations to the national health services.^5^ In conclusion, our findings of high prevalence estimates for major mental health and substance use disorders among prisoners in LMICs present an important global mental health challenge, indicate a treatment gap, and might raise concerns about human rights violations.

## References

[1] Walmsley R (2016).

[2] Cohen JE, Amon JJ (2008). Health and human rights concerns of drug users in detention in Guangxi Province, China. PLoS Med.

[3] Almanzar S, Katz CL, Harry B (2015). Treatment of mentally ill offenders in nine developing Latin American countries. J Am Acad Psychiatry Law.

[4] Fazel S, Seewald K (2012). Severe mental illness in 33 588 prisoners worldwide: systematic review and meta-regression analysis. Br J Psychiatry.

[5] Jack HE, Fricchione G, Chibanda D, Thornicroft G, Machando D, Kidia K (2018). Mental health of incarcerated people: a global call to action. Lancet Psychiatry.

[6] Andreoli SB, Dos Santos MM, Quintana MI (2014). Prevalence of mental disorders among prisoners in the state of Sao Paulo, Brazil. PLoS One.

[7] Mundt AP, Alvarado R, Fritsch R (2013). Prevalence Rates of Mental Disorders in Chilean Prisons. PLoS One.

[8] Fazel S, Yoon IA, Hayes AJ (2017). Substance use disorders in prisoners: an updated systematic review and meta-regression analysis in recently incarcerated men and women. Addiction.

[9] Baranyi G, Cassidy M, Fazel S, Priebe S, Mundt AP (2018). Prevalence of posttraumatic stress disorder in prisoners. Epidemiol Rev.

[10] Gates ML, Turney A, Ferguson E, Walker V, Staples-Horne M (2017). Associations among substance use, mental health disorders, and self-harm in a prison population: examining group risk for suicide attempt. Int J Environ Res Public Health.

[11] Spittal MJ, Forsyth S, Borschmann R, Young JT, Kinner SA (2017). Modifiable risk factors for external cause mortality after release from prison: a nested case-control study. Epidemiol Psychiatr Sci.

[12] Baillargeon J, Binswanger IA, Penn JV, Williams BA, Murray OJ (2009). Psychiatric disorders and repeat incarcerations: the revolving prison door. Am J Psychiatry.

[13] Moher D, Shamseer L, Clarke M (2015). Preferred reporting items for systematic review and meta-analysis protocols (PRISMA-P) 2015 statement. Syst Rev.

[14] Tavares GP, Scheffer M, Martins de Almeida RM (2012). Drugs, violence and emotional aspects in prisoners. Psicol Reflex Crit.

[15] Fido AA, al-Jabally M (1993). Presence of psychiatric morbidity in prison population in Kuwait. Ann Clin Psychiatry.

[16] Munn Z, Moola S, Riitano D, Lisy K (2014). The development of a critical appraisal tool for use in systematic reviews addressing questions of prevalence. Int J Health Policy Manag.

[17] Kelley GA, Kelley KS (2012). Statistical models for meta-analysis: a brief tutorial. World J Methodol.

[18] Nyaga VN, Arbyn M, Aerts M (2014). Metaprop: a Stata command to perform meta-analysis of binomial data. Arch Public Health.

[19] Higgins JP, Thompson SG, Deeks JJ, Altman DG (2003). Measuring inconsistency in meta-analyses. BMJ.

[20] Fazel S, Hayes AJ, Bartellas K, Clerici M, Trestman R (2016). Mental health of prisoners: prevalence, adverse outcomes, and interventions. Lancet Psychiatry.

[21] Jackson D, Law M, Rucker G, Schwarzer G (2017). The Hartung-Knapp modification for random-effects meta-analysis: a useful refinement but are there any residual concerns?. Stat Med.

[22] Higgins JPT, Green S (2011). Cochrane handbook for systematic reviews of interventions version 5.1.0. Updated March 2011.

[23] Beijer U, Wolf A, Fazel S (2012). Prevalence of tuberculosis, hepatitis C virus, and HIV in homeless people: a systematic review and meta-analysis. Lancet Infect Dis.

[24] Egger M, Davey Smith G, Schneider M, Minder C (1997). Bias in meta-analysis detected by a simple, graphical test. BMJ.

[25] Nanéma D, Goumbri P, Karfo K, Ouango JG, Ouédraogo A (2014). Epidemiological and clinical aspects of mental disorders in prisons in Ouagadougou, Burkina Faso. Annales Africaines de Psychiatrie.

[26] Pondé MP, Freire AC, Mendonca MS (2011). The prevalence of mental disorders in prisoners in the city of Salvador, Bahia, Brazil. J Forensic Sci.

[27] Canazaro D, de Lima Argimon II (2010). Characteristics, depressive symptoms, and associated factors in incarcerated women in the State of Rio Grande do Sul, Brazil. Cad Saude Publica.

[28] Silva NC, Rosa MI, Amboni G, Mina F, Comim CM, Quevedo J (2011). Psychiatric disorders and risk factors in a prison population. ACM arq catarin med.

[29] Mundt AP, Kastner S, Larraín S, Fritsch R, Priebe S (2016). Prevalence of mental disorders at admission to the penal justice system in emerging countries: a study from Chile. Epidemiol Psychiatr Sci.

[30] El-Gilany A, Khater M, Gomaa Z, Hussein E, Hamdy I (2016). Psychiatric disorders among prisoners: a national study in Egypt. East Asian Arch Psychiatry.

[31] Ayirolimeethal A, Ragesh G, Ramanujam JM, George B (2014). Psychiatric morbidity among prisoners. Indian J Psychiatry.

[32] Goyal SK, Singh P, Gargi PD, Goyal S, Garg A (2011). Psychiatric morbidity in prisoners. Indian J Psychiatry.

[33] Kumar V, Daria U (2013). Psychiatric morbidity in prisoners. Indian J Psychiatry.

[34] Math SB, Murthy P, Parthasarathy R, Kumar CD, Madhusudhan S (2011).

[35] Joshi P, Kukreja S, Desousa A, Shah N, Shrivastava A (2014). Psychopathology and other contributing stressful factors in female offenders: an exploratory study. Indian J Forensic Med Toxicol.

[36] Assadi SM, Noroozian M, Pakravannejad M (2006). Psychiatric morbidity among sentenced prisoners: prevalence study in Iran. Br J Psychiatry.

[37] Zamzam R, Hatta SM (2000). Specific psychiatric disorders among convicted female offenders in a Malaysian prison. Malaysian J Psychiatry.

[38] Adesanya A, Ohaeri JU, Ogunlesi AO, Adamson TA, Odejide OA (1997). Psychoactive substance abuse among inmates of a Nigerian prison population. Drug Alcohol Depend.

[39] Majekodunmi O, Obadeji A, Oluwole L, Oyelami R (2017). Depression in prison population: Demographic and clinical predictors. J Forensic Sci Med.

[40] Naidoo S, Mkize DL (2012). Prevalence of mental disorders in a prison population in Durban, South Africa. Afr J Psychiatry.

[41] Ndetei D, Khasakhala L, Mutiso V, Harder V (2008).

[42] Niriella MA, Hapangama A, Luke HP, Pathmeswaran A, Kuruppuarachchi KA, de Silva HJ (2015). Prevalence of hepatitis B and hepatitis C infections and their relationship to injectable drug use in a cohort of Sri Lankan prison inmates. Ceylon Med J.

[43] Salifou S, Wenkourama D, Soedje KMA, Anagonou L, Agouda B, Dassa KS (2018). Mental health of women detained in the civil prison of Lome. Health Sci Dis.

[44] Boşgelmez S, Aker T, Oznur AK, Ford JD (2010). Assessment of lifetime history of exposure to traumatic stressors by incarcerated adults with the turkish version of the traumatic events screening instrument for adults (TESI-A): A pilot study. J Trauma Dissociation.

[45] Kaya N, Guler O, Cilli AS (2004). Prevalence of psychiatric disorders among prisoners in Konya prison [in Turkish]. Anadolu Psikiyatri Dergisi.

[46] Steel Z, Marnane C, Iranpour C (2014). The global prevalence of common mental disorders: a systematic review and meta-analysis 1980-2013. Int J Epidemiol.

[47] Saha S, Chant D, Welham J, McGrath J (2005). A systematic review of the prevalence of schizophrenia. PLoS Med.

[48] Mundt AP, Baranyi G, Gabrysch C, Fazel S (2018). Substance use during imprisonment in low- and middle-income countries. Epidemiol Rev.

[49] Bennett T, Holloway K (2009). The causal connection between drug misuse and crime. Br J Criminol.

[50] Walker J, Illingworth C, Canning A (2014). Changes in mental state associated with prison environments: a systematic review. Acta Psychiatr Scand.

[51] Baier A, Fritsch R, Ignatyev Y, Priebe S, Mundt AP (2016). The course of major depression during imprisonment—a one year cohort study. J Affect Disord.

[52] Yoon IA, Slade K, Fazel S (2017). Outcomes of psychological therapies for prisoners with mental health problems: a systematic review and meta-analysis. J Consult Clin Psychol.

[53] Hopkin G, Evans-Lacko S, Forrester A, Shaw J, Thornicroft G (2018). Interventions at the transition from prison to community for prisoners with mental illness: a systematic review. Adm Policy Ment Health.

[54] Shidhaye R, Murhar V, Gangale S (2017). The effect of VISHRAM, a grass-roots community-based mental health programme, on the treatment gap for depression in rural communities in India: a population-based study. Lancet Psychiatry.

[55] Naslund JA, Aschbrenner KA, Araya R (2017). Digital technology for treating and preventing mental disorders in low-income and middle-income countries: a narrative review of the literature. Lancet Psychiatry.

[56] de Jesus MJ, Razzouk D, Thara R, Eaton J, Thornicroft G (2009). Packages of care for schizophrenia in low- and middle-income countries. PLoS Med.

