# Supplementary material for: Severe mental illness and substance use disorders in prisoners in low-income and middle-income countries: a systematic review and meta-analysis of prevalence studies
Source: Lancet Glob Health. 2019 Mar 14;7(4):e461–71. doi: 10.1016/S2214-109X(18)30539-4 (PMC6419715; doi:10.1016/S2214-109X(18)30539-4)
Supplement: Supplementary appendix [file mmc1.pdf]

THE LANCET
Global Health

Supplementary appendix

This appendix formed part of the original submission and has been peer reviewed. We post it as supplied by the authors.

Supplement to: Baranyi G, Scholl C, Fazel S, Patel V, Priebe S, Mundt AP. Severe mental illness and substance use disorders in prisoners in low-income and middle-income countries: a systematic review and meta-analysis of prevalence studies.
Lancet Glob Health 2019; **7:** e461–71.

SUPPLEMENTARY MATERIALS

Supplementary Material 1: General search terms

Supplementary Table 1: Database searches

Supplementary Material 2: Critical Appraisal Tool

Supplementary Table 2: Quality appraisal of studies reporting severe mental disorders or substance use disorders in prison populations of low- and middle-income countries

Supplementary Table 3: Meta-regression analysis of study characteristics relating to severe mental illnesses

Supplementary Table 4: Sensitivity analysis for severe mental illnesses

Supplementary Table 5: Meta-regression analysis of study characteristics relating to substance use disorders

Supplementary Figure 1: Funnel Plots estimating small sample bias in studies reporting severe mental illnesses and substance use disorders in prison samples in low- and middle-income countries

Supplementary Material 1: General search terms

1. Prison condition:
prison* OR imprison* OR sentence* OR remand* OR jail* OR correctional OR “criminal justice”

2. Epidemiological studies:
Epidemiology (Subject Heading) OR prevalence OR incidence

3. Mental health outcome:
Mental Disorders (Subject Heading) OR Substance-Related Disorders (Subject Heading) OR psychiatr* OR psycho* OR depress* OR bipolar OR schizophren* OR alcohol* OR substance OR addict*

Limit:
1987 to Present

Supplementary Table 1: Database searches

Prison	Epidemiology	Mental health
Applied Social Sciences Index and Abstracts (ASSIA) (1/5/2018) (373)[*]		
ab(Prison* or imprison* or sentence* or remand* or jail* or correctional or criminal justice) (21,955)	ab(prevalence OR incidence) OR su.Exact("epidemiology") (44,092)	Exact("drug abuse" OR "alcohol related disorders" OR "substance abuse" OR "mental disorders") OR ab(psychiatr* or psycho* or depress* or bipolar or schizophren* or alcohol* or substance or addict*) (176,787)
CAB Abstracts 1973 to 2018 Week 16 (1/5/2018) (112)[*]		
(Prison* or imprison* or sentence* or remand* or jail* or correctional or criminal justice).mp (2,164)	epidemiology/ or (prevalence or incidence).mp. (447,300)	mental disorders/ or substance abuse/ or (psychiatr* or psycho* or depress* or bipolar or schizophren* or alcohol* or substance or addict*).mp. (285,944)
China Academic Journals Full-text Database in China National Knowledge Infrastructure (CNKI) (24/7/2018)[†]		
prison AND mental; prison AND psychiatric; prison AND depression; prison AND psychosis; prison AND substance; prison AND abuse; prison AND dependence; prison AND addiction<br>jail AND mental; jail AND psychiatric; jail AND depression; jail AND psychosis; jail AND substance; jail AND abuse; jail AND dependence; jail AND addiction		
Criminal Justice Database (1/5/2018) (342)[*]		
all(Prison* OR imprison* OR sentence* OR remand* OR jail* OR correctional OR “criminal justice”) (51,735)	Exact("epidemiology") OR ab(prevalence or incidence) (10,989)	Exact("substance-related disorders" OR "mental disorders") OR ab(psychiatr* or psycho* or depress* or bipolar or schizophren* or alcohol* or substance or addict*) (63,735)
Embase 1980 to 2018 Week 18 (1/5/2018) (2,718)[*]		
(Prison* or imprison* or sentence* or remand* or jail* or correctional or criminal justice).mp. (58,898)	epidemiology/ or (prevalence or incidence).mp. (1,909,979)	mental disease/ or drug dependence/ or alcoholism/ or (psychiatr* or psycho* or depress* or bipolar or schizophren* or alcohol* or substance or addict*)mp. (2,863,150)
Global Health 1973 to 2018 Week 16 (1/5/2018) (516)[*]		
(Prison* or imprison* or sentence* or remand* or jail* or correctional or criminal justice).mp (5,029)	epidemiology/ or (prevalence or incidence).mp. (474,935)	mental disorders/ or substance abuse/ or (psychiatr* or psycho* or depress* or bipolar or schizophren* or alcohol* or substance or addict*).mp. (222,205)
International Bibliography of the Social Sciences (IBSS) (1/5/2018) (116)[*]		
ab(Prison* OR imprison* OR sentence* OR remand* OR jail* OR correctional OR “criminal justice”) (21,927)	Exact("epidemiology") OR ab(prevalence OR incidence) (30,465)	Exact("substance-related disorders" OR "mental disorders") OR ab(psychiatr* or psycho* or depress* or bipolar or schizophren* or alcohol* or substance or addict*) (66,384)
Latin American and Caribbean Health Sciences Literature (LILACS) (1/5/2018) (120)[*]		

(tw:(prison$ OR imprison$ OR sentence$ OR remand$ OR jail$ OR correctional OR criminal justice) (1,484)	(tw:(epidemiolog$ OR prevalence OR incidence) (111.772)	(tw:(mental or psychiatr$ or psycho$ or depress$ or bipolar or schizophren$ or alcohol$ or substance or addict$) (108.175)
MEDLINE(R) Epub Ahead of Print, In-Process & Other Non-Indexed Citations, Ovid MEDLINE(R) Daily and Ovid MEDLINE(R) 1946 to Present (1/5/2018) (2,150)*		
(Prison* or imprison* or sentence* or remand* or jail* or correctional or criminal justice).mp. (46,923)	EPIDEMIOLOGY/ or (prevalence or incidence).mp. (1,272,070)	Mental Disorders/ or Substance-Related Disorders/ or (psychiatr* or psycho* or depress* or bipolar or schizophren* or alcohol* or substance or addict*).mp. (1,272,070)
National Criminal Justice Reference Service (NCJRS) (1/5/2018) (40)*		
ab(Prison* or imprison* or sentence* or remand* or jail* or correctional or "criminal justice") (63,463)	ab(epidemiolog* OR prevalence OR incidence) (9,603)	Exact("substance abuse (60399)" OR "mental disorders (04529)") AND ab(psychiatr* or psycho* or depress* or bipolar or schizophren* or alcohol* or substance or addict*) (1,183)
PAIS Index (1/5/2018) (27)*		
ab(Prison* or imprison* or sentence* or remand* or jail* or correctional or "criminal justice") (8,867)	Exact("epidemiology") OR ab(prevalence OR incidence) (11,016)	Exact("substance-related disorders" OR "mental disorders") OR ab(psychiatr* or psycho* or depress* or bipolar or schizophren* or alcohol* or substance or addict*) (13,484)
PsycINFO 1806 to April Week 4 2018 (1/5/2018) (1,811)*		
(Prison* or imprison* or sentence* or remand* or jail* or correctional or criminal justice).mp. (77,669)	exp EPIDEMIOLOGY/or (prevalence or incidence).mp. [mp=title, abstract, heading word, table of contents, key concepts, original title, tests & measures] (149,748)	exp Mental Disorders/ exp Addiction/ or exp Alcohol Abuse/ or exp Drug Abuse/ or exp Drug Addiction/ or exp Alcoholism/ or exp Drug Dependency/or (psychiatr* or psycho* or depress* or bipolar or schizophren* or alcohol* or substance or addict*).mp. (1,807,188)
Scopus (1/5/2018) (4,125)*		
TITLE-ABS-KEY (prison* OR imprison* OR sentence* OR remand* OR jail* OR correctional OR "criminal justice") (157,693)	TITLE-ABS-KEY (epidemiolog* OR prevalence OR incidence) (2,313,318)	TITLE-ABS-KEY (mental OR psychiatr* OR psycho* OR depress* OR bipolar OR schizophren* OR alcohol* OR substance OR addict*) (4,607,258)
Social Services Abstracts (1/5/2018) (33)*		
ab(Prison* OR imprison* OR sentence* OR remand* OR jail* OR correctional OR “criminal justice”) (7,132)	Exact("epidemiology") OR ab(prevalence OR incidence) (15,613)	Exact("substance-related disorders" OR "mental disorders") AND ab(psychiatr* or psycho* or depress* or bipolar or schizophren* or alcohol* or substance or addict*) (4,275)
**Grey literature**		
Open Grey (1/5/2018) (27)†		
prison* OR imprison* OR sentence* OR remand* OR jail* OR correctional OR “criminal justice” (3,587)	epidemiolog* OR prevalence OR incidence (14,287)	mental OR substance OR psychiatr* OR psycholog* OR smok* OR alcohol* OR substance OR drug OR addict* OR inject* (74,044)
ProQuest Dissertations & Theses Global (1/5/2018) (210)*		
ab(Prison* OR imprison* OR sentence* OR remand* OR jail* OR correctional OR “criminal justice”) (31,175)	Exact("epidemiology") OR ab(prevalence OR incidence) (70,689)	Exact("drug abuse" OR "alcohol use" OR "alcoholism" OR "drug addiction" OR "mental disorders") OR ab(psychiatr* or psycho* or depress* or bipolar or schizophren* or alcohol* or substance or addict*) (174,767)
Google Scholar (2/5/2018)† ‡		
prison* OR imprison* OR sentence* OR remand* OR jail* OR correctional OR “criminal justice”	prevalence OR incidence	psychiatr* OR psycho* OR substance OR depressi* OR bipolar

* Number after discarding duplicates.
† Hits were not exported in reference manager.
‡ Searches were separately run for each LMIC and about the first 20% of hits were screened.

Supplementary Material 1: Critical Appraisal Tool[*]

Reviewer: ____________________ Date: __________________

Author: ____________________ Year: _______ Record No. _______

No.	Item	Rating
**External Validity**		
1.	Was the study's target population a close representation of the national population in relation to relevant variables? [†]<br>1 point: Samples from more than one institution, representative for a province or the whole country.<br>0 point: Otherwise.	
2.	Was the sample representative of the target population?[*]<br>1 point: Stratified random, random, population or systematic sampling.<br>0 point: Convenience sampling.	
3.	Was the sample size adequate?[*]<br>1 point: Sample size equal to or greater than 200 participants or a pre-calculated sample size reached.<br>0 point: Otherwise.	
4.	Was the data analysis conducted with sufficient coverage of the identified sample?[*]<br>1 point: Participation rate was equal or higher than 75% or a non-response analysis yielded non-significant results.<br>0 point: Otherwise.	
**Internal Validity**		
5.	Were the study subjects and the setting described in detail?[*]<br>1 point: Reporting of the descriptive statistics to describe the sample included gender AND at least three other socio-demographic or criminal characteristic.<br>0 point: Otherwise.	
6.	Was the same standardized interview used for data collection by all subjects? [†]<br>1 point: Yes.<br>0 point: Other mode of data collection were applied.	
7.	Were objective, standard criteria used for the measurement of the condition?[*]<br>1 point: Disorders were assessed according to the criteria of DSM or ICD<br>0 point: Conditions were measured with standardized screening scale, which did not fulfil disorder criteria.	
8.	Was the condition measured reliably?[*]<br>1 point: Psychiatrist(s)/psychologist(s) made the diagnoses.<br>0 point: Trained interviewer(s) made the diagnoses.	
9.	Was there appropriate statistical reporting?[*]<br>1 point: Confidence intervals/standard deviations were calculated for prevalence rates.<br>0 point: Only prevalence estimates were reported.	
10.	Were subpopulations identified using objective criteria?[*]<br>1 point: The study explored only female/only male prisoners OR the results were reported separately for male/female prisoners.<br>0 point: The study explored both male and female prisoners, but results were not reported separately.	
	**Score:**	

[*] taken or adapted from JBI Critical Appraisal Checklist for Studies Reporting Prevalence [1]
† taken or adapted from the Risk of Bias Tool [2]

Supplementary Table 2: Quality appraisal of studies reporting severe mental disorders or substance use disorders in prison populations of low- and middle-income countries

Study	1.	2.	3.	4.	5.	6.	7.	8.	9.	10.	Score
Adesanya et al.[3]	No	Yes	Yes	Yes	Yes	Yes	Yes	No	No	No	6
Andreoli et al.[4] M	Yes	Yes	Yes	No	Yes	Yes	Yes	No	Yes	Yes	8
Andreoli et al.[4] F	Yes	Yes	Yes	Yes	Yes	Yes	Yes	No	Yes	Yes	9
Assadi et al.[5]	No	Yes	Yes	Yes	Yes	Yes	Yes	Yes	Yes	Yes	9
Ayirolimeethal et al.[6] M	No	Yes	Yes	Yes	Yes	Yes	Yes	Yes	No	Yes	8
Ayirolimeethal et al.[6] F	No	Yes	No	Yes	Yes	Yes	Yes	Yes	No	Yes	7
Boşgelmez et al.[7] M	No	Yes	No	Yes	Yes	Yes	Yes	Yes	No	Yes	7
Boşgelmez et al.[7] F	No	Yes	No	Yes	Yes	Yes	Yes	Yes	No	Yes	7
Canazaro & Argimon[8]	No	Yes	Yes	Yes	Yes	Yes	Yes	Yes	No	Yes	8
El-Gilany et al.[9]	Yes	Yes	Yes	Yes	Yes	Yes	Yes	Yes	No	No	8
Goyal et al.[10]	No	Yes	Yes	Yes	Yes	Yes	Yes	Yes	No	No	7
Joshi et al. [11]	No	Yes	No	No	Yes	Yes	Yes	Yes	No	Yes	6
Kaya et al.[12]	No	Yes	Yes	Yes	Yes	Yes	Yes	No	No	No	6
Kumar & Daria[13]	No	Yes	Yes	Yes	Yes	Yes	Yes	Yes	No	No	7
Majekodunmi et al.[14]	No	Yes	Yes	Yes	Yes	Yes	Yes	Yes	No	Yes	8
Math et al.[15]	No	Yes	Yes	No	Yes	No	Yes	No	No	No	4
Mundt et al.[16] M	Yes	Yes	Yes	Yes	Yes	Yes	Yes	No	Yes	Yes	9
Mundt et al.[16] F	Yes	Yes	No	Yes	Yes	Yes	Yes	No	Yes	Yes	8
Mundt et al.[17] M	Yes	Yes	Yes	Yes	Yes	Yes	Yes	Yes	Yes	Yes	10
Mundt et al.[17] F	Yes	Yes	No	Yes	Yes	Yes	Yes	Yes	Yes	Yes	9
Naidoo & Mkize[18]	No	Yes	Yes	Yes	Yes	Yes	Yes	Yes	No	No	7
Nanéma et al.[19]	No	Yes	Yes	Yes	Yes	Yes	Yes	No	No	No	6
Ndetei et al.[20]	No	Yes	No	No	Yes	Yes	Yes	Yes	No	No	5
Niriella et al.[21] M	Yes	Yes	Yes	Yes	No	Yes	Yes	No	No	Yes	7
Niriella et al.[21] F	Yes	Yes	No	Yes	No	Yes	Yes	No	No	Yes	6
Pondé et al.[22]	Yes	Yes	Yes	Yes	No	Yes	Yes	No	No	Yes	7
Salifou et al.[23]	No	Yes	No	Yes	Yes	Yes	Yes	Yes	No	Yes	7
Silva et al.[24] M	No	Yes	Yes	Yes	Yes	Yes	Yes	No	No	Yes	7
Silva et al.[24] F	No	Yes	No	Yes	Yes	Yes	Yes	No	No	Yes	6
Zamzam & Hatta[25]	No	Yes	No	Yes	Yes	Yes	Yes	Yes	No	Yes	7

F=Female, M=Male

Supplementary Table 3: Meta-regression analysis of study characteristics relating to severe mental illnesses

	One-year prevalence of psychotic disorders				One-year prevalence of major depressive disorders			
	**ß**	**95% CI**		**p**	**ß**	**95% CI**		**p**
		**lower**	**upper**			**lower**	**upper**	
UNIVARIATE ANALYSIS								
Sampled from all prisoners vs at intake	0·186	0·117	0·255	**<0·001**	0·199	0·066	0·333	**0·005**
Sex (male vs female)	-0·007	-0·100	0·084	0·862	0·047	-0·081	0·175	0·459
Age (cont.)	-0·014	-0·035	0·007	0·179	-0·001	-0·031	0·032	0·962
Sample size (n<500 vs n≥500)	-0·076	-0·125	-0·027	**0·004**	-0·116	-0·227	-0·006	**0·039**
Time spent in prison (<1 y vs ≥1 y)	-0·146	-0·205	-0·087	**<0·001**	-0·091	-0·226	0·044	0·172
Year of data collection (cont.)	0·002	-0·008	0·011	0·716	0·008	-0·007	0·022	0·305
Interviewer (mental health professional vs other)	0·043	-0·006	0·093	0·083	0·107	-0·005	0·219	0·061
Diagnostic criteria (DSM vs ICD)	-0·052	-0·129	0·024	0·165	-0·054	-0·189	0·081	0·413
Non-response rate (cont.)	-0·0001	-0·003	0·003	0·967	-0·0005	-0·006	0·005	0·835
Quality of the sample (cont.)	0·002	-0·020	0·025	0·831	0·026	-0·013	0·065	0·189
WHO-regions	$F_{(5,16)}$ = 0·55			0·733	$F_{(5,20)}$ = 0·40			0·840
MULTIVARATE ANALYSIS								
Sampled from all prisoners vs at intake	0·138	0·019	0·257	**0·026**	0·168	0·033	0·303	**0·017**
Sample size (n<500 vs n≥500)	-0·015	-0·104	0·074	0·720	-0·078	-0·182	0·026	0·134
Time spent in prison (<1 y vs ≥1 y)	-0·040	-0·170	0·090	0·515				

Bold values are significant (p<0·05).

Supplementary Table 4: Sensitivity analysis of severe mental illnesses among prisoner populations in low- and middle-income countries (without studies conducted at intake to prison)

	Prevalence Rate		Prevalence Ratio	
	12-month	6-month	12-month	6-month
Psychotic illnesses	6·2 (4·0-8·6)	4·5 (3·1-6·2)	10·7 (6·7-17·1)	13·4 (7·4-24·3)
male	6·6 (3·7-10·2)	4·9 (3·1-7·1)	11·1 (6·0-20·4)	14·4 (6·7-30·7)
female	5·7 (1·9-11·0)	2·7 (0·4-6·3)	8·3 (5·0-13·6)	11·8 (4·5-30·9)
Major Depression	16·0 (11·7-20·8)	12·6 (7·9-18·0)	5·3 (3·9-7·2)	5·2 (3·6-7·6)
male	15·9 (11·1-21·4)	13·3 (8·2-19·4)	6·3 (4·4-9·1)	6·1 (4·0-9·5)
female	19·4 (11·7-28·5)	15·2 (5·1-29·0)	5·3 (3·1-8·9)	6·7 (3·6-12·3)
Schizophrenia			7·9 (4·9-12·7)	
male			7·0 (4·1-11·9)	
female			7·2 (3·9-13·2)	

Supplementary Table 5: Meta-regression analysis of study characteristics relating to substance use disorders

	Current alcohol use disorders				Current drug use disorders				Lifetime drug use disorders			
	ß	95% CI		p	ß	95% CI		p	ß	95% CI		p
		lower	upper			lower	upper			lower	upper	
Sex (male vs female)	-0·018	-0·127	0·091	0·718	-0·014	-0·096	0·068	0·704	0·078	-0·227	0·383	0·579
Age (cont.)	-0·009	-0·043	0·026	0·581	0·015	-0·014	0·045	0·265	0·009	-0·027	0·046	0·572
Sample size (n<500 vs n≥500)	0·036	-0·050	0·122	0·369	-0·037	-0·104	0·030	0·242	-0·238	-0·479	0·003	0·052
Time spent in prison (<1 y vs ≥1 y)	-0·0002	-0·189	0·189	0·998	0·020	-0·367	0·326	0·891	-0·108	-0·951	0·735	0·740
Year of data collection (cont.)	0·006	-0·008	0·021	0·343	-0·001	-0·008	-0·007	0·807	-0·008	-0·052	0·035	0·654
Interviewer (mental health professional vs other)	-0·031	-0·139	0·077	0·539	0·056	-0·049	0·162	0·254	0·181	-0·095	0·457	0·172
Diagnostic criteria (DSM vs ICD)	0·033	-0·056	0·122	0·422	-0·051	-0·120	0·017	0·122	-0·154	-0·450	0·141	0·263
Non-response rate (cont.)	-0·001	-0·003	0·002	0·531	-0·002	-0·005	0·001	0·214	-0·004	-0·021	0·014	0·623
Quality of the study (cont.)	-0·024	-0·035	-0·013	**0·001**	0·002	-0·027	0·032	0·875	0·081	-0·005	0·166	0·062
WHO-regions*		$F_{(4,7)}$ = 7·47		**0·011**		$F_{(4,6)}$ = 0·51		0·733		$F_{(4,6)}$ = 3·75		0·073
Africa	0·036	-0·120	0·191	0·604					-			
Americas	0·030	-0·102	0·163	0·607					0·171	-0·208	0·551	0·311
Eastern Mediterranean	Ref.								0·627	0·146	1·108	**0·019**
Europe	-								Ref.			
South-East Asia	0·140	0·010	0·271	**0·038**					0·091	-0·292	0·475	0·581
Western Pacific	0·025	-0·267	0·317	0·845					0·371	-0·165	0·909	0·141

Bold values are significant (p<0·05).

*Region with the lowest pooled prevalence estimate is taken as reference category.

Supplementary Figure 1: Funnel Plots estimating small sample bias in studies reporting severe mental illnesses and substance use disorders in prison populations in low- and middle-income countries

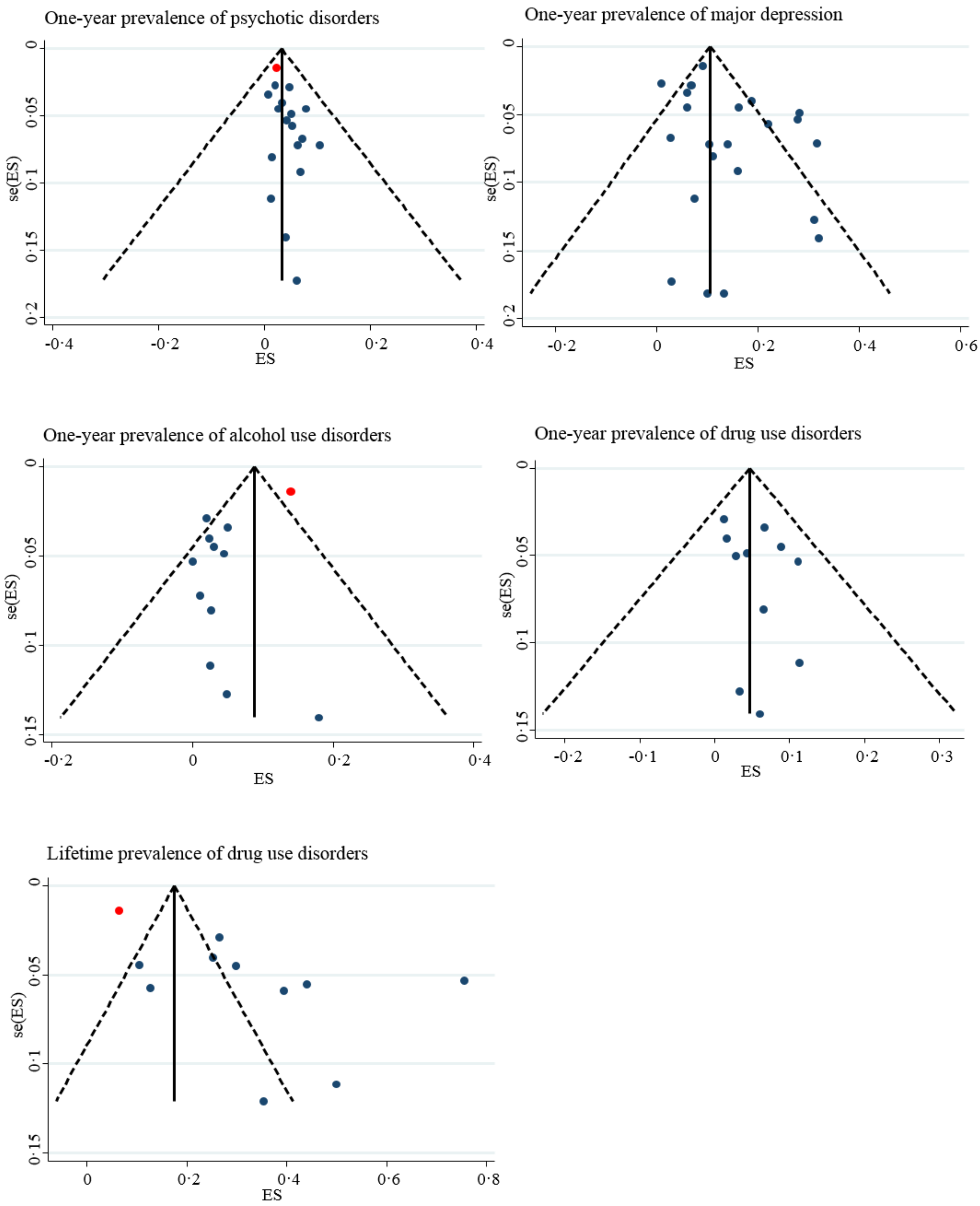

Estimates are plotted against the standard error of the estimate; red point symbolize estimates excluded for sensitivity analysis.

References

1. Munn Z, Moola S, Riitano D, Lisy K. The development of a critical appraisal tool for use in systematic reviews addressing questions of prevalence. *Int J Health Policy Manag* 2014; **3**: 123–8.
2. Hoy D, Brooks P, Woolf A, et al. Assessing risk of bias in prevalence studies: modification of an existing tool and evidence of interrater agreement. *J Clin Epidemiol* 2012; **65**: 934–9.
3. Adesanya A, Ohaeri JU, Ogunlesi AO, Adamson TA, Odejide OA. Psychoactive substance abuse among inmates of a Nigerian prison population. *Drug Alcohol Depend* 1997; **47**: 39–44.
4. Andreoli SB, Dos Santos MM, Quintana MI, et al. Prevalence of mental disorders among prisoners in the state of Sao Paulo, Brazil. *PLoS ONE* 2014; **9**: e88836.
5. Assadi SM, Noroozian M, Pakravannejad M, et al. Psychiatric morbidity among sentenced prisoners: Prevalence study in Iran. *Br J Psychiatry* 2006; **188**: 159–64.
6. Ayirolimeethal A, Ragesh G, Ramanujam JM, George B. Psychiatric morbidity among prisoners. *Indian J Psychiatry* 2014; **56**: 150–3.
7. Boşgelmez S, Aker T, Oznur AK, Ford JD. Assessment of lifetime history of exposure to traumatic stressors by incarcerated adults with the turkish version of the traumatic events screening instrument for adults (TESI-A): A pilot study. *J Trauma Dissociation* 2010; **11**: 407–23.
8. Canazaro D, de Lima Argimon II. Characteristics, depressive symptoms, and associated factors in incarcerated women in the State of Rio Grande do Sul, Brazil [in Portuguese]. *Cad Saude Publica* 2010; **26**: 1323–33.
9. El-Gilany A, Khater M, Gomaa Z, Hussein E, Hamdy I. Psychiatric Disorders among Prisoners: A National Study in Egypt. *East Asian Arch Psychiatry* 2016; **26**: 30–8.
10. Goyal SK, Singh P, Gargi PD, Goyal S, Garg A. Psychiatric morbidity in prisoners. *Indian J Psychiatry* 2011; **53**: 253–7.
11. Joshi P, Kukreja S, Desousa A, Shah N, Shrivastava A. Psychopathology and other contributing stressful factors in female offenders: An exploratory study. *Indian Journal of Forensic Medicine and Toxicology* 2014; **8**: 149–55.
12. Kaya N, Guler O, Cilli AS. Prevalence of psychiatric disorders among prisoners in Konya prison [in Turkish]. *Anadolu Psikiyatri Dergisi* 2004; **5**: 85–91.
13. Kumar V, Daria U. Psychiatric morbidity in prisoners. *Indian J Psychiatry* 2013; **55**: 366–70.
14. Majekodunmi O, Obadeji A, Oluwole L, Oyelami R. Depression in prison population: Demographic and clinical predictors. *J Forensic Sci Med* 2017; **3**: 122–7.
15. Math SB, Murthy P, Parthasarathy R, Kumar CD, Madhusudhan S. Mental Health and Substance Use Problems in Prisons. The Bangalore Prison Mental Health Study: Local Lessons for National Action. Bangalore, India: National Institute of Mental Health and Neuro Sciences; 2011.
16. Mundt AP, Alvarado R, Fritsch R, et al. Prevalence Rates of Mental Disorders in Chilean Prisons. *PLoS ONE* 2013; **8**: e69109.
17. Mundt AP, Kastner S, Larraín S, Fritsch R, Priebe S. Prevalence of mental disorders at admission to the penal justice system in emerging countries: A study from Chile. *Epidemiol Psychiatr Sci* 2016; **25**: 441–9.
18. Naidoo S, Mkize DL. Prevalence of mental disorders in a prison population in Durban, South Africa. *Afr J Psychiatry* 2012; **15**: 30–5.
19. Nanéma D, Goumbri P, Karfo K, Ouango JG, Ouédraogo A. Epidemiological and clinical aspects of mental disorders in prisons in Ouagadougou, Burkina Faso [in French]. *Annales Africaines de Psychiatrie* 2014; **3**: 59–75.
20. Ndetei D, Khasakhala L, Mutiso V, Harder V. Mental disorders and HIV risk behaviors among prisoners in South Sudan. Nairobi, Kenya: United Nations Office On Drugs And Crime (UNODC), 2008.
21. Niriella MA, Hapangama A, Luke HP, Pathmeswaran A, Kuruppuarachchi KA, de Silva HJ. Prevalence of hepatitis B and hepatitis C infections and their relationship to injectable drug use in a cohort of Sri Lankan prison inmates. *Ceylon Med J* 2015; **60**: 18–20.
22. Pondé MP, Freire AC, Mendonca MS. The prevalence of mental disorders in prisoners in the city of Salvador, Bahia, Brazil. *J Forensic Sci 2011*; **56**: 679–82.
23. Salifou S, Wenkourama D, Soedje KMA, Anagonou L, Agouda B, Dassa KS. Mental health of women detained in the civil prison of Lome [in French]. *Health Sci Dis* 2018; **19**: 46–50.
24. Silva NC, Rosa MI, Amboni G, Mina F, Comim CM, Quevedo J. Psychiatric disorders and risk factors in a prison population [in Portuguese]. *ACM arq catarin med* 2011; **40**: 72–6.
25. Zamzam R, Hatta SM. Specific Psychiatric Disorders Among Convicted Female Offenders in a Malaysian Prison. *Malaysian Journal of Psychiatry* 2000; **8**: 34–42.
